# XASDB: a new database of experimental interactive X-ray absorption spectra

**DOI:** 10.1107/S1600577525006174

**Published:** 2025-07-31

**Authors:** Xueqi Song, Haodong Yao, Fei Zhan, Shuguang Chen, Lirong Zheng, Chenyan Ma, Lei Zheng, Haifeng Zhao

**Affiliations:** a Multi-Disciplinary Research Division Institute of High Energy Physics 19B Yuquan Road, Shijingshan District Beijing100049 People’s Republic of China; b Institute of High Energy Physics University of Chinese Academy of Sciences 19A Yuquan Road, Shijingshan District Beijing100049 People’s Republic of China; University of Essex, United Kingdom

**Keywords:** X-ray absorption spectroscopy, database, XASMatch

## Abstract

We present a dedicated and comprehensive database for experimental X-ray absorption spectroscopy (XAS) data, an integrated and user-friendly platform that combines spectrum visualization, raw data processing, spectrum matching and downloading, thereby offering a holistic solution for understanding XAS data. Leveraging the unique nature of XAS data, we have designed the MySQL table structure and developed professional plotting tools to present the data. Furthermore, the database – named XASDB – incorporates a variety of data processing tools, including data normalization as well as flexible searching and downloading options. A toolkit called XASMatch is integrated in the platform, enabling users to identify the most similar spectra within the database to their input spectra and rank with scores. An API interface is also provided to facilitate sharing data with other XAS databases and users. A total of 152 spectra measured at Beijing Synchrotron Radiation Facility for metals, oxides and minerals standards are included in the database. Applications integrated with artificial intelligence are advancing, since more high solution spectra with new standards will be available after operation of a high energy photon source to be commissioned at the end of the year.

## Abstract 2

A comprehensive database for experimental X-ray absorption spectroscopy data is presented. This integrated and user-friendly platform combines spectrum visualization, raw data processing, spectrum matching and downloading.

## 1. Introduction

X-ray absorption spectroscopy (XAS) (Rehr & Albers, 2000 ▸) is a principal technique for material characterization, offering critical information about the local atomic chemical environment of interested species (Guo *et al.*, 2023 ▸), including bond length, charge transfer, symmetry of coordination atoms and core/valence bands. XAS is particularly significant for the analysis of solid materials, especially those that are amorphous, disordered or multicomponent. Due to its broad range of applications across various scientific fields, XAS has emerged as an essential tool for research in areas such as catalytic reactions and battery materials (Gaur *et al.*, 2023 ▸).

In XAS experiments, reference materials are standardized samples of known structure and chemical state that are used to calibrate instruments, verify the reliability of experimental data, and compare and analyze the data with those of the samples to be tested. Their core role is to provide benchmark information to help researchers accurately analyze the local structure, elemental valence and coordination environment of the samples to be tested, and other key parameters. The analysis of reference materials is a fundamental aspect of the data analysis process, particularly for XAS experiments. Beamline users and the broader XAS community can significantly benefit from a reliable and extensive database of reference sample spectra obtained under standardized and well characterized experimental conditions. XAS data serve as a foundation for users to facilitate data comparisons and to enhance their understanding and knowledge of XAS techniques and applications. Large databases can mitigate the potential for misinterpretation and enhance our understanding of XAS data. Beamline users and the XAS community, in general, stand to gain substantially from the availability of a comprehensive and reliable spectral reference base. There are several XAS databases available now for users, despite the challenges associated with constructing a database (Asakura *et al.*, 2018 ▸).

The XASLIB database (https://xaslib.xrayabsorption.org), developed and maintained by the Center for Advanced Photon Sources at the University of Chicago, USA, is specifically dedicated to XAS data. Currently, the database contains 277 data items covering 20 elements, with most of the experimental data collected from multiple synchrotron beamlines in USA. XASLIB offers a flexible and efficient search and filtering mechanism that enables users to conduct precise searches based on a variety of criteria, including elements, suites, samples and beamlines. Furthermore, the database features robust visualization and download capabilities, allowing users to view and download single or multiple spectra easily. Additionally, XASLIB provides users with the ability to evaluate and upload data.

SSHADE (https://www.sshade.eu/), co-hosted by OSUG Data Center and Université des Alpes de Grenoble, France, also provides access to XAS spectra, an interoperable solid spectroscopy database infrastructure that provides spectral and photometric data obtained by various spectroscopic techniques over the whole electromagnetic spectrum. The database is extensive, containing a total of 595 entries of XAS data. SSHADE offers robust search capabilities, allowing users to search by keyword and perform complex queries using filters. Additionally, the website provides the ability to plot individual spectra, and the images support regional zooming. Users can select a single spectrum for download.

The Materials Data Repository (MDR; https://mdr.nims.go.jp/) is a data repository for hosting materials research data and publications, created by Masashi Ishii *et al.* (Ishii *et al.*, 2023 ▸). MDR collects and hosts not only papers and presentations but also materials data. Currently, MDR hosts a significant amount of data, including 2264 entries of X-ray absorption fine structure (XAFS) data. The website provides users with filter searches and keyword searches, as well as the ability to plot and download individual spectra.

The Canadian Light Source also manage a database (https://xasdb.lightsource.ca/) dedicated to XAS data. The database provides a periodic table of elements for users to search by element. After obtaining the search results, users are able to batch plot the spectra, which can be zoomed in and out, and they can download individual experimental spectra. It is worth mentioning that the database is continuously updated and provides a variety of data processing functions, including data normalization, Fourier transform and energy-to-wavevector (*E*–*k*) (Iglesias-Juez *et al.*, 2022 ▸) transformation.

RefXAS (http://xafsdb.ddns.net) is a specialized database for XAS data, currently consisting of 23 spectra and constructed by Sebastian Paripsa *et al.* (Paripsa *et al.*, 2024 ▸). RefXAS is continually updated to provide a range of features. The website offers search functions by element and keyword; it also provides users with raw, normalized, χ(*k*) and χ(*R*) plots for individual spectra. The database supports users in uploading and downloading data. It is worth mentioning that RefXAS has established a set of quality criteria to assess the quality of XAS data.

Overall, these XAS databases provide powerful tools for the development of the XAS community. They offer users a wealth of XAS data, means to search by element or qualification, and advanced visualization tools. Users are also able to perform a range of data processing functions on XAS data. However, these websites have some limitations in terms of functionality; for example, some websites only provide simple search functions and cannot perform complex searches. Additionally, some websites provide raw XAS data but do not include data processing functions. Furthermore, some websites only offer the ability to plot and download XAS data for a single entry and cannot perform batch operations. Lastly, some websites do not provide the regional zoom function for the spectrum plotting tool.

We provide here an alternative experimental XAS database (https://xasdb.ihep.ac.cn/), established MySQL table structure, and implementing a variety of features based on a user-friendly user interface design. The database provides two methods of searching: element-based and qualification-based. It includes a powerful visualization tool for XAS data, options for single or batch downloads of XAS data, and normalization methods based on buckling backgrounds. The database places high value on data interaction with users—a toolkit named XASMatch is proved to assist users in identifying similar spectra in the database by inputting their spectrum; these similar spectra are accompanied by consistency scores. We aim to promote sharing and exchange of data within the field of XAS, thereby fostering progress and innovation in research.

## 2. Construction of XASDB

The construction of XASDB includes the design of the database architecture, the design of the MySQL table structure, the data functionality and the data statistics.

### 2.1. Database architecture

**Figure 1:**
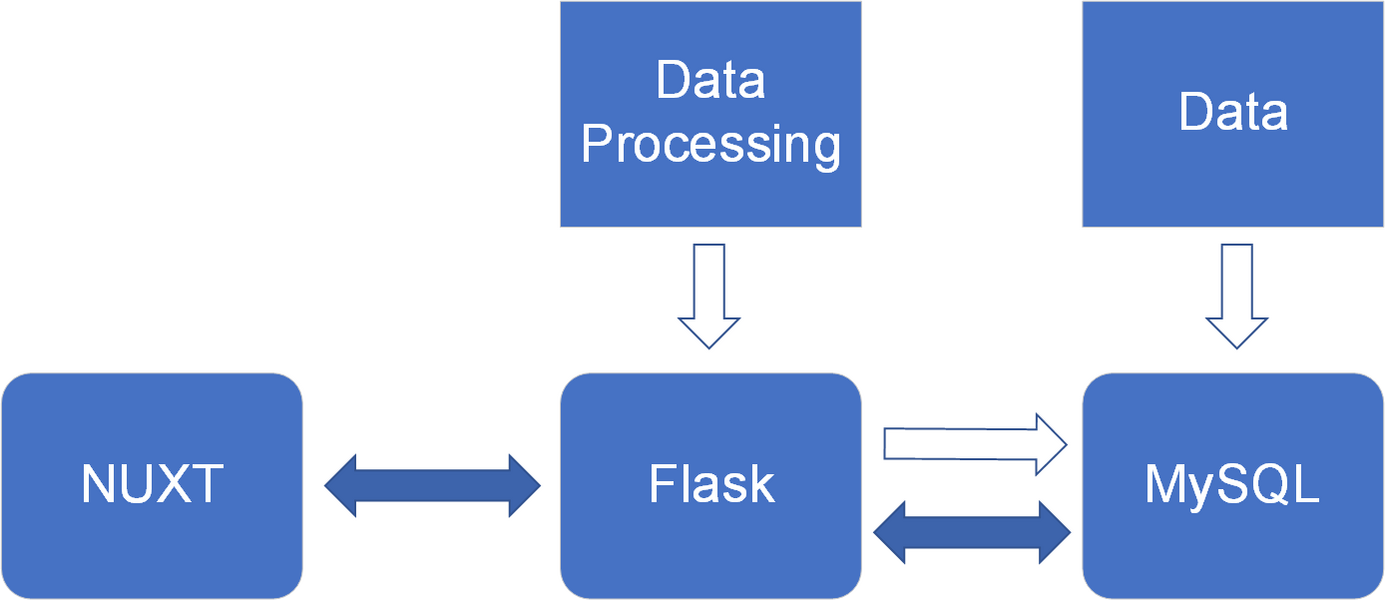
XASDB architecture diagram, using NUXT as the front-end framework, Flask as the back-end framework, the data are stored in the MySQL database, but also then back-end data processing.

The database we developed is presented through a website that uses a decoupled architecture, which separates front-end and back-end components. To ensure the stability of the website, the front-end uses the powerful and flexible NUXT framework (https://nuxt.com), while the back-end uses the general and lightweight Flask framework (Dwyer *et al.*, 2017 ▸) (https://flask.palletsprojects.com/). Due to the universality and flexibility of MySQL (DuBois, 2013 ▸) (https://www.mysql.com/) database technology, similar to most databases, the experimental XAS data storage uses MySQL. The overall architecture of the website is shown in Fig. 1 ▸. The user interacts with the front-end web pages, and the web interface sends requests to the back-end after executing commands. The back-end receives the request, processes the request, interacts with the database to retrieve the requested data, and then sends the data back to the user interface as a response, thus providing the user with the required information. In addition, the back-end can perform data processing and store the results in bulk in the database.

### 2.2. MySQL table structure

**Figure 2:**
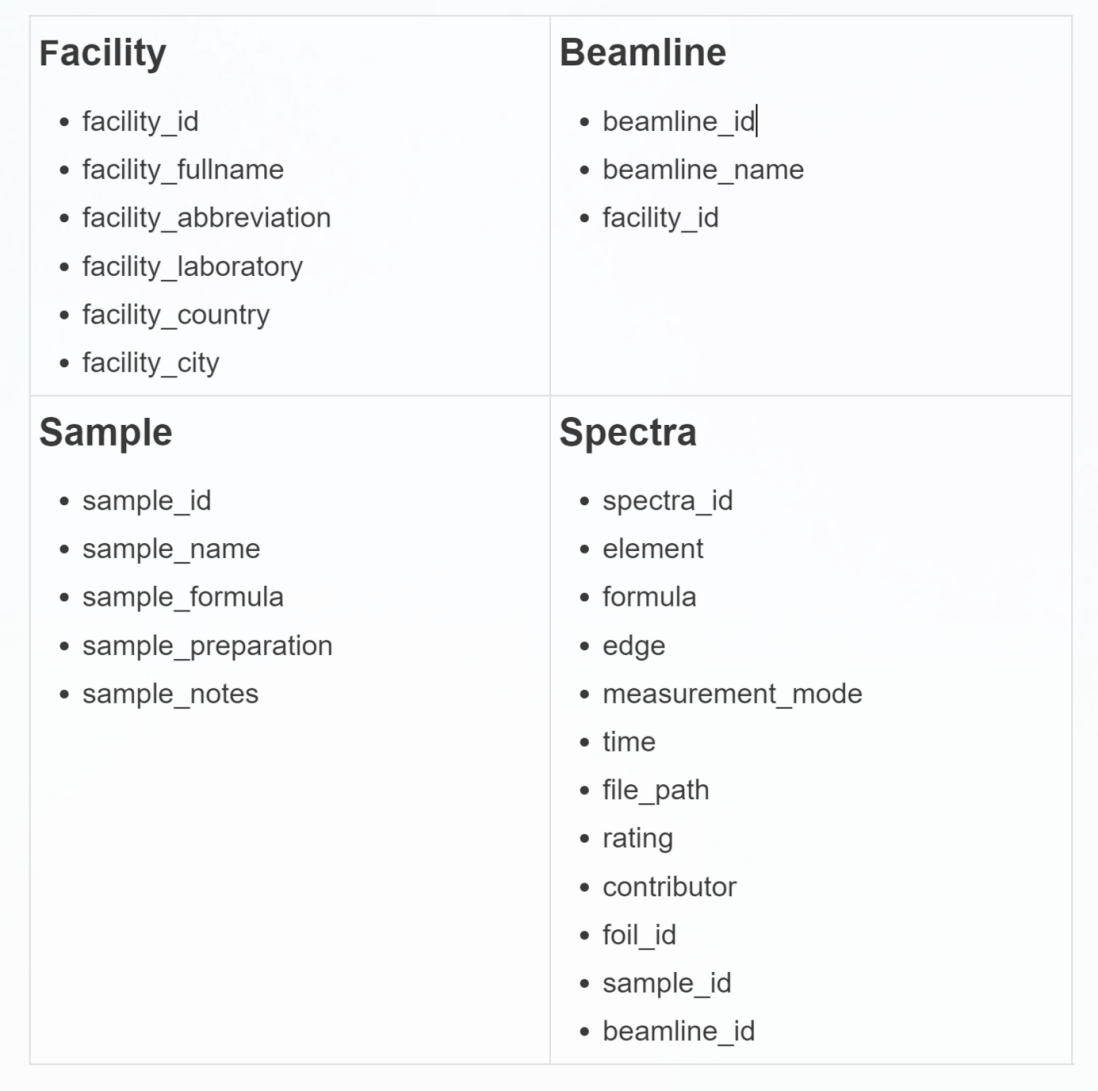
MySQL database table structure in XASDB, consisting of four tables: Facility, Beamline, Sample and Spectra.

Table structure design is very important when building a database—it is the core of MySQL. Fig. 2 ▸ shows the design of the table structure of the MySQL database that we built. It consists of four main components: Facility, Beamline, Sample and Spectra. The Facility table records the full name of the synchrotron radiation device, its abbreviation, the laboratory it is affiliated with, its country and its city. The Beamline table provides detailed information about the name of the beamline and the associated synchrotron radiation device. The Sample table includes the sample name, its chemical formula, preparation information and any relevant notes. The Spectra table documents the absorbing elements of the absorption spectrum, molecular formula, absorption edge, measurement mode, time, storage path, scoring level and donor of the absorption spectrum. Collectively, these four tables are designed to establish a comprehensive database structure for effective data management and utilization.

### 2.3. Data functions

#### 2.3.1. Website

**Figure 3:**
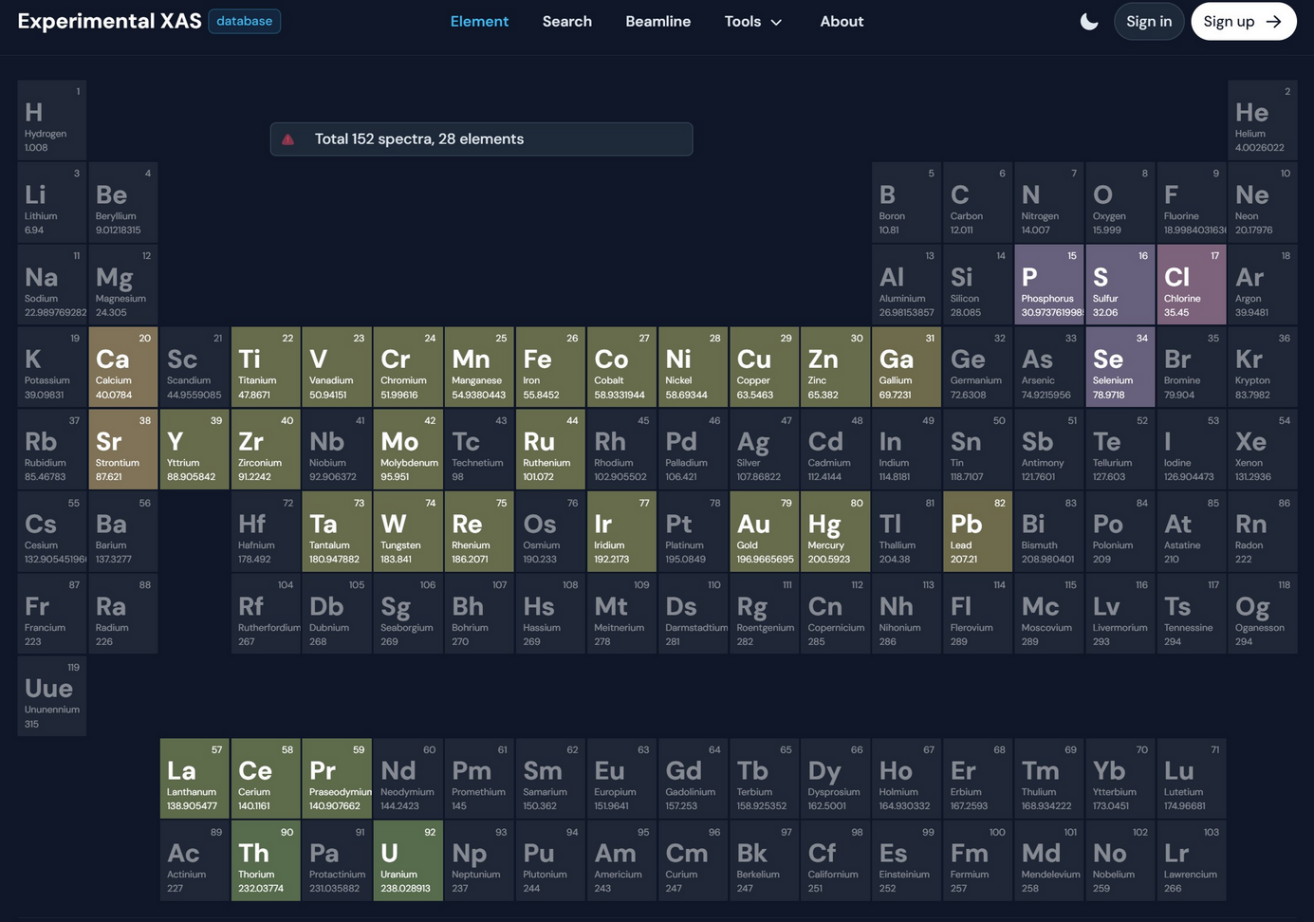
Screenshot of the homepage of XASDB, providing a quick view of the XAS data, encoded in the form of the periodic table of elements.

The URL of the database is https://xasdb.ihep.ac.cn, and the homepage of the website is presented in Fig. 3 ▸. It consists of five main components, namely the periodic table search page, the qualification search page, the beamline statistics page, the tools page and the about page. Within this structure, the tools page provides a spectroscopic matching tool, XASMatch. For more information about the use of the website, refer to the user manual on the website’s about page (https://xasdb.ihep.ac.cn/about).

#### 2.3.2. Data visualization

**Figure 4:**
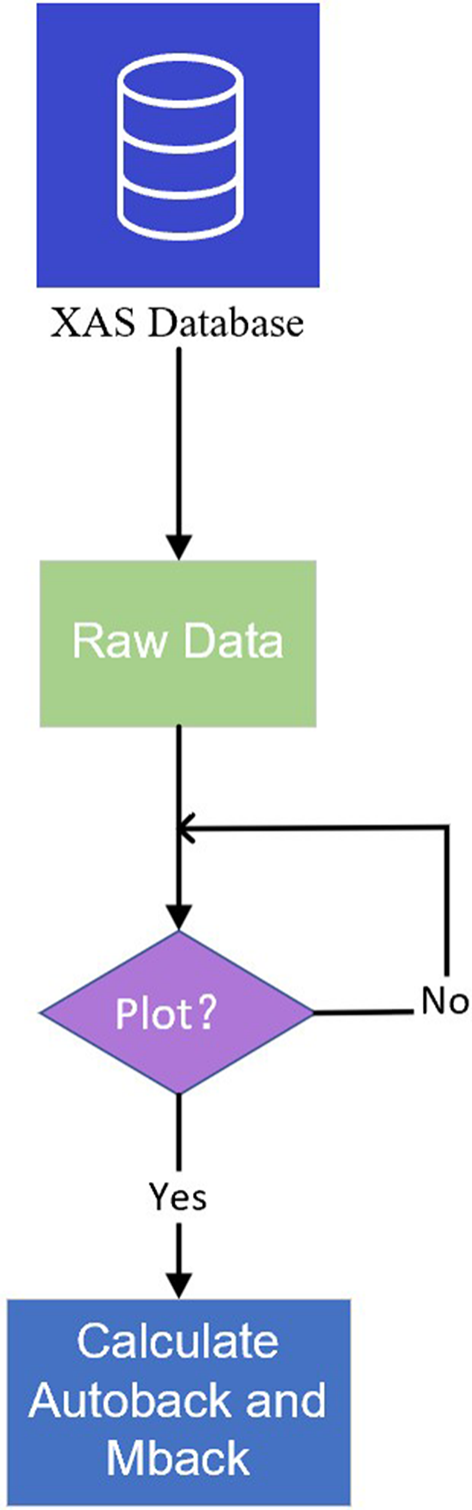
Data flow diagram for data visualization in XASDB.

As presented in Fig. 4 ▸, a flowchart illustrating the data visualization process in this study is provided. On the website platform, users are equipped with powerful interactive data manipulation capabilities to conduct in-depth and diverse comparative analyses of the data. Specifically, users can interactively compare XAS data for different substances or different absorption edges.

**Figure 5:**
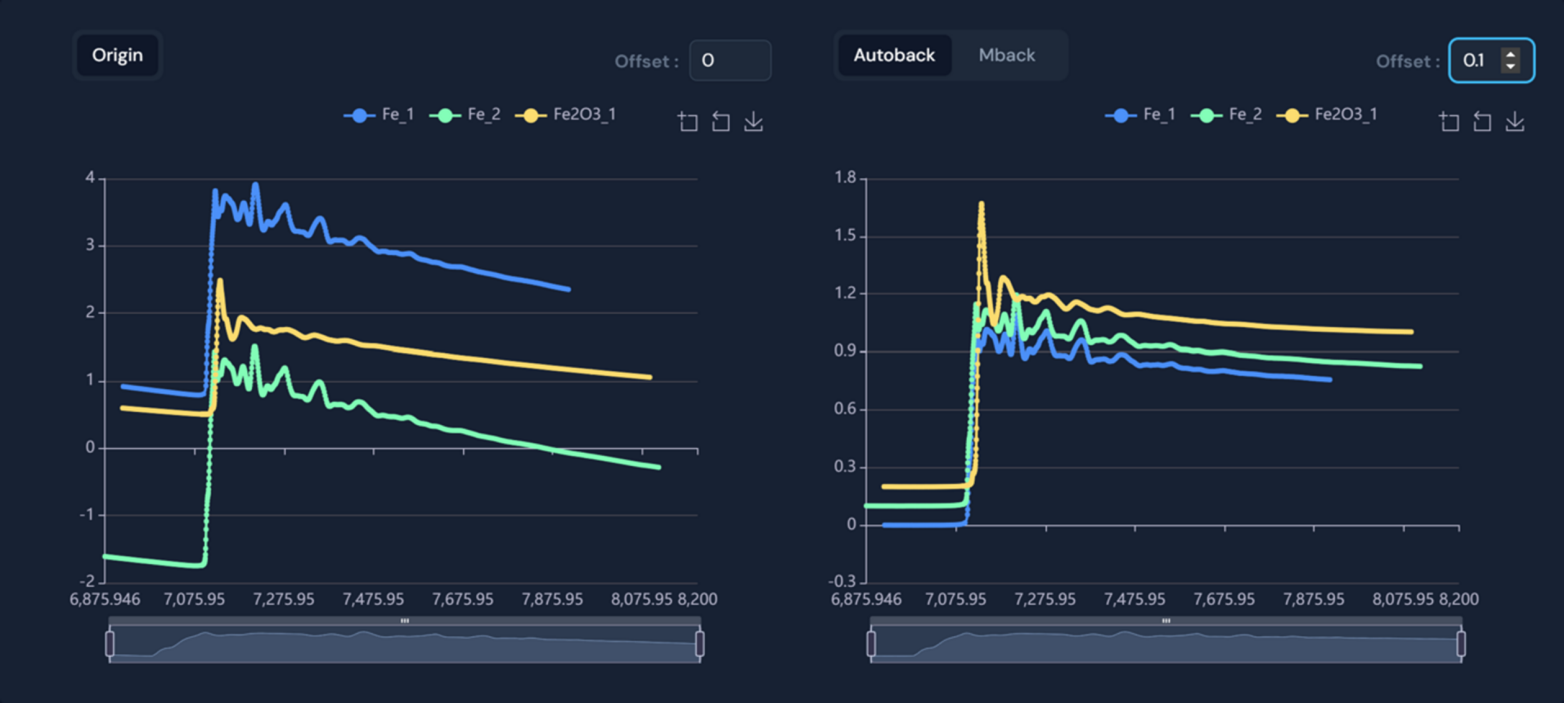
Screenshot of *K*-edge XAS spectra of three iron-containing samples selected from XASDB. The left panel shows the original plots of the three XAS spectra, while the right one shows the processed spectra after background subtraction and normalization. An offset of 0.1 is set for an optimal view, as shown in the upper right corner of the figure.

In terms of data processing flexibility, users can select the required data through batch checking and transform it into intuitive visualizations. For example, when users select specific data such as Fe_1, Fe_2, Fe2O3_1 *etc*. from a large dataset for visualization, the corresponding XAS spectra are displayed in different colors, as illustrated in Fig. 5 ▸.

When users select the data with specified absorption spectra for visualization, the website provides both Autoback and Mback algorithms for background subtraction and normalizing to raw data, while retaining the pre-edge, post-edge and processed data in preparation for visualization. Autoback uses the pre_edge (https://xraypy.github.io/xraylarch/xafs_preedge.html#the-pre-edge-function) function provided by Larch (Newville, 2013 ▸). Mback (Weng *et al.*, 2005 ▸) is a program developed for normalizing X-ray absorption data to tabulated mass absorption coefficients. Larch provides an implementation of the Mback (https://xraypy.github.io/xraylarch/xafs_preedge.html#the-mback-algorithm) algorithm with an option of using the modification proposed by Lee *et al.* (2009 ▸). The left panel of Fig. 4 ▸ displays the original data, while the right panel shows the data processed using the Autoback algorithm; data processed by the Mback algorithm can be displayed when selected.

Furthermore, the website is equipped with a professional drawing tool that integrates advanced area scaling and offsetting capabilities, which enhances the user’s experience during data comparison and analysis.

#### 2.3.3. Data download

The database is publicly accessible and aims to provide open and convenient access to and utilization of data for all users. We are committed to building an open and transparent database platform that all users interested in XAS can browse, query and utilize the data resources. Currently, we offer two flexible and user-friendly methods for downloading data, aimed at providing users with diverse options for accessing information. The first one allows users to select and download a single data entry, which is suitable for those who need specific information and wish to quickly and conveniently obtain it. The second one permits users to batch-select the data of interest, subsequently packaging the selected data into a compressed file for download. This approach is advantageous for users who require large volumes of data or multiple data files, thereby enhancing the efficiency and convenience of the downloading process. At the same time, we have added a regular inspection mechanism to ensure the stability and reliability of the user download function.

#### 2.3.4. XASMatch

**Figure 6:**
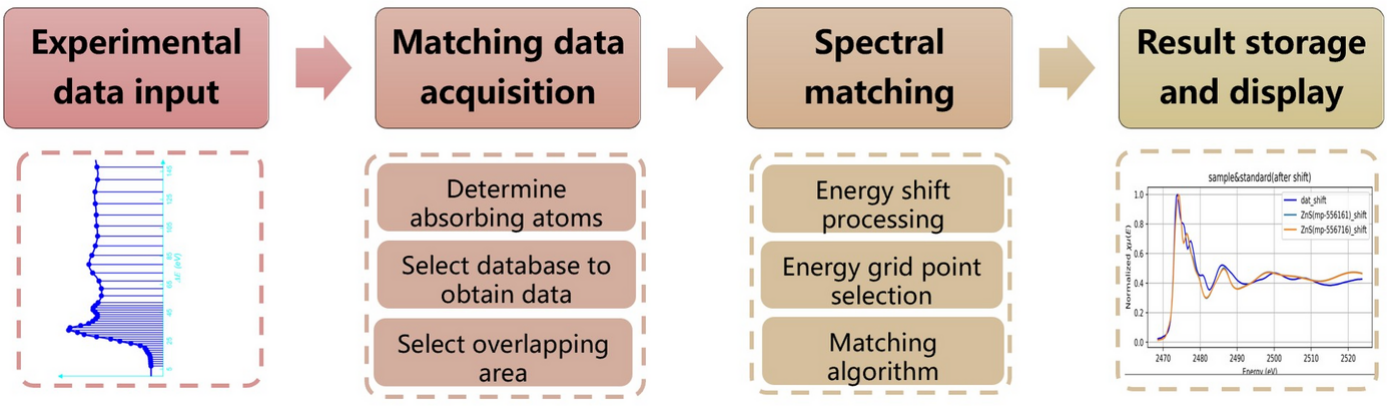
Roadmap of the XASMatch spectral matching workflow: experimental XAS spectra acquisition; absorbing atom identification, database filtering and spectral overlap region selection; energy shift calibration and application of matching algorithms; score and optimal reference spectra visualization.

The website provides XASMatch, an interactive spectral matching tool developed in-house, to conduct precise analysis and matching of XAS spectra. XASMatch enables researchers to import experimental XAS spectra and accurately determine the absorption edge (E0) through the integrated Larch (Newville, 2013 ▸) module’s find_e0() function, identifying the absorbing element and corresponding edge (workflow illustrated in Fig. 6 ▸). Researchers can upload experimental XAS spectrum data in two formats: dat and txt. The uploaded data must contain two columns: energy and mu. In the future, we will add support for more data types. The ‘Data Security Policy’ published on the about page (https://xasdb.ihep.ac.cn/about) explains our unpublished raw data uploaded by users. The system will automatically and completely delete it after completing the real-time analysis. Researchers can then conveniently select reference spectra from either a local database or the Materials Project theoretical database (Jain *et al.*, 2013 ▸) (https://next-gen.materialsproject.org/). Moreover, XASMatch provides advanced filtering options, allowing the inclusion or exclusion of specific elements and specifying the number of elements (greater than, equal to or less than a given count) to meet specific research criteria. Notably, when selecting the theoretical database from Materials Project, an additional filter based on structural stability (by default considering structures with E above_hull less than 0.01 eV) is available, ensuring higher reliability of the reference data.

After selecting the reference database, XASMatch offers three methods for energy alignment: alignment based on the absorption edge position (E0), spectral alignment at a characteristic 6% jump of the absorption peak, and alignment based on the white-line peak position. Particularly, the 6% jump alignment method, using the intersection of the absorption spectrum with a horizontal line at 6% of the peak height, reduces the influence of noise. Following spectral alignment, select the overlapping energy region for similarity calculation. Recognizing that different energy intervals carry varying spectral importance, XASMatch not only provides traditional uniform sampling but also incorporates logarithmic sampling, emphasizing lower-energy spectral regions, thus highlighting important early stage spectral features.

For similarity evaluation, XASMatch integrates multiple advanced spectral matching algorithms, including least squares, area difference, length of curves, dynamic time warping, Euclidean distance, Pearson correlation coefficient, Manhattan distance, Minkowski distance, and cosine similarity. By default, the Pearson correlation coefficient is utilized for calculating similarity scores, consistent with its successful application by Zheng *et al.* (2018 ▸) in their development of the Ensemble-Learned Spectra IdEntification (ELSIE) algorithm in XASDB.

**Figure 7:**
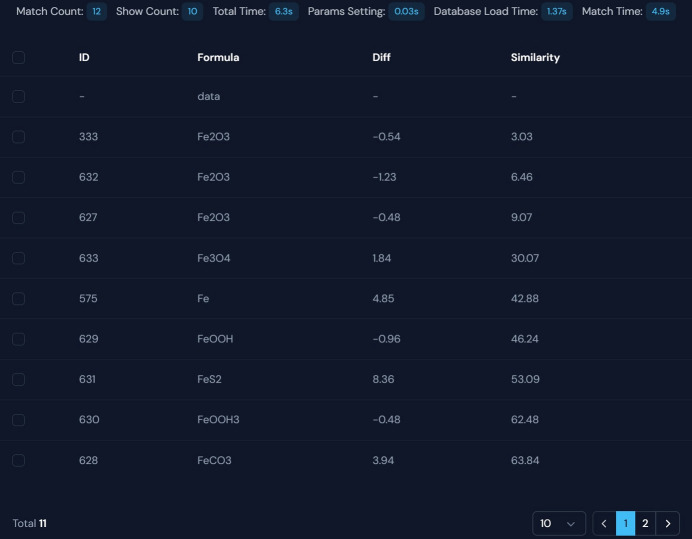
Screenshot of the XASMatch output interface after matching the Fe_2_O_3_ spectrum, displaying key parameters (count, time) and ranked reference spectra.

An illustrative example is presented by matching the experimental Fe_2_O_3_ spectrum, as shown in Fig. 7 ▸. XASMatch quickly generates comprehensive results, clearly displaying key parameters such as the total matching time (6.3 s), including parameter setting (0.03 s), database loading (1.37 s) and the actual matching process (4.8 s). The results, sorted from highest to lowest similarity, clearly demonstrate the efficiency of the tool. Researchers can conveniently inspect these outcomes, analyze spectral differences through the presented similarity rankings, and easily export both data and figures, enhancing the efficiency and accuracy of the XAS match.

### 2.4. Statistics

**Figure 8:**
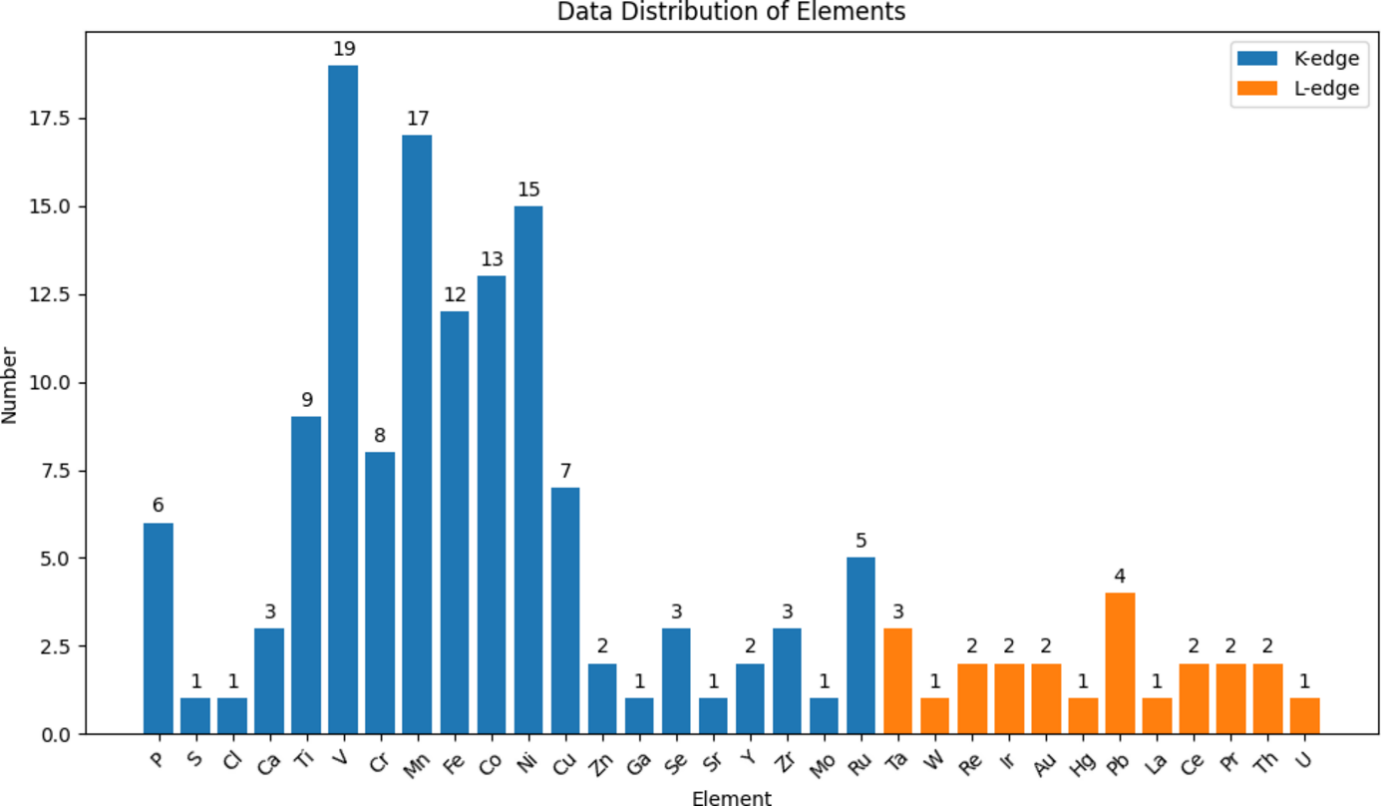
Statistical chart of XAS data in XASDB.

We provide a statistical chart for the spectra in the database as shown in Fig. 8 ▸. It provides a quick view for the data accepted to fully understand the synthesis of the data in XASDB. As of March 2025, the statistics of experimental XAS data in this database website are as follows:

Total number of absorption spectra: 152.

Total number of absorption edges: *K*-edge: 129; *L*_3_-edge: 23.

Total number of elements: 32.

There are a total of 152 samples, including 89 molecular formulas. We can see that *K*/*L*-edges dominate the experimental XAS data that are included in XASDB, as in most other XAS library/databases present.

## 3. Data

All the experimental data included in XASDB have been measured at the beamlines 1W1 and 4B7A at Beijing Synchrotron Radiation Facility (BSRF), collected in transmission or fluorescence mode by scientists at beamlines or users over decades. The data from BSRF’s experimental stations are in a unified txt format. In the future, more data will be obtained from the High Energy Photon Source (HEPS) and will use a unified HDF5 format. These data in XASDB are derived from standard sample data from experimental stations, which adhere to the FAIR (Wilkinson *et al.*, 2016 ▸) principles.

**Figure 9:**
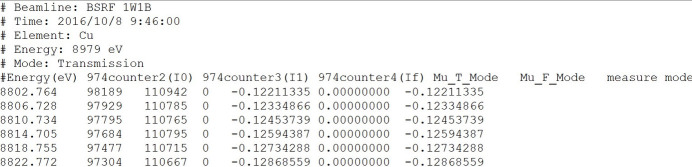
Example diagram of metadata stored in XASDB.

We performed quality checks on the experimental data in XASDB. The entire data screening and quality check process is as follows: first, we use the find_e0 function provided by Larch to find e0, then we check the table to determine its absorption edge, and finally we perform manual screening for confirmation. Considering the energy transfer issue, we encourage users to report any problems they encounter during use and provide us with feedback and suggestions. The common metadata of data are extracted and shown with a plot of the spectrum line, as shown in Fig. 9 ▸. Users could have a quick impression of the labels of data, where and when it is collected, which mode it is applied, the energy range, and so on. Moreover, they serve as keywords for matching in the advanced search provided in the portal of XASDB.

## 4. Interface

In order to enrich the content of the website and help users with advanced processing, we designed interfaces following the principles of RESTful API (Patni, 2017 ▸), which facilitates communication between the front-end and the back-end. RESTful API is an architecture based on the HTTP protocol, and the core component is the Uniform Resource Locator (URL) (Le *et al.*, 2018 ▸), and we maintained a clear hierarchical structure when designing the URLs in order to improve readability. This interface structure design lays the foundation for interconnecting our website with other websites, making data sharing possible. Currently, through this interface, the International XAFS DB Portal (https://ixdb.jxafs.org/) in Japan can search our data, thus realizing data sharing and contributing to the XAS community.

## 5. Conclusions

We have established an XAS database, adopting a front-end and back-end separated architecture, and designed the MySQL table structure accordingly. In terms of data visualization, we equipped users with professional drawing tools that enable them to compare and analyze XAS data. Regarding data processing, we provided users with two data normalization methods—Autoback and Mback—and a spectral matching tool—XASMatch. For data download, users can flexibly choose to download either single datasets or batch data. In terms of data acquisition, we offer users an interface API. Currently, the data in this database are exclusively derived from BSRF, but more high-quality data would be included from the beamlines of HEPS, which is in the top few high brightness, high energy synchrotron radiation facilities in the world, available for users at the end of this year. At the same time, we are actively cooperating with other databases, and XASDB is listed in the International XAFS Database Portal (https://ixdb.jxafs.org/), thus further enhancing information sharing.

To ensure the long-term sustainability and development of the XAS database, we will enhance potential data processing functions, such as Fourier transform and wavelet transform. Furthermore, to enrich the content of the database, we will develop a series of XAS data quality standards to facilitate the uploading of XAS data and subsequent quality assessment by users. In recent years, the rapid development of artificial intelligence has made datasets an essential component of neural network training. Therefore, we plan to integrate the database with artificial intelligence technologies and develop application programming interfaces (APIs) to enable machine learning applications to access the data, thereby further accelerating the interpretation of XAS data.

The advancement of the XAS database will be continued, with a commitment to providing support for the management and analysis of XAS data being maintained. It is hoped that this initiative will be developed into a powerful tool for promoting the rapid growth of the XAS community, thereby benefiting all members. Furthermore, it is anticipated that this work will further promote the progress and innovation of scientific research while strengthening scientific exchanges and cooperation within the XAS field.
